# A Case of Neurotrophic Keratopathy Concomitant to Brain Metastasis

**DOI:** 10.7759/cureus.2309

**Published:** 2018-03-12

**Authors:** Chiara Bonzano, Elisabetta Bonzano, Carlo Alberto Cutolo, Riccardo Scotto, Carlo Enrico Traverso

**Affiliations:** 1 Clinica Oculistica, University of Genoa Ospedale Policlinico San Martino; 2 Radiation Oncology, University of Genoa Ospedale Policlinico San Martino; 3 Clinica Oculistica, University of Genoa, Ospedale Policlinico San Martino

**Keywords:** autologous serum, radiation therapy, brain metastasis, neurotrophic keratitis, trigeminal neuropathy

## Abstract

We report a case of a 63-year-old Caucasian female referred to the cornea service of Clinica Oculistica with a neurotrophic corneal ulcer, decreased corneal sensitivity, absent corneal reflex, and decreased lacrimation. The medical record review was relevant for mastectomy and adjuvant therapy for breast cancer complicated by pontocerebellar angle metastasis. Eye patching and application of antibiotic and vitamin ointments were prescribed at first, without a significant improvement. Thus, treatment with autologous serum was started. In about two weeks, the cornea recovered and visual acuity improved with a residual corneal scarring. Finally, we should mention that, in our case, the main cause of the neurotrophic corneal ulcer could be identified in the previous trigeminal damage at the pontocerebellar angle and trigeminal ganglion. Sensory nerves play an important regulatory role via neuro-mediators on corneal wound healing, as denervation may interfere with cellular metabolism and inhibit mitosis, leading to an epithelial defect even with no direct damage.

## Introduction

Neurotrophic keratitis (NK) is a rare corneal disease characterized by a prevalence of less than 5/10,000 in the general population [1]. Damage at any level of the trigeminal nerve from its cranial nucleus to the corneal nerve endings can cause corneal sensitivity reduction, spontaneous epithelium breakdown, and impairment of corneal healing leading to the development of NK [2]. Clinical presentation may be insidious because patients with NK rarely complain of symptoms, except for blurred vision due to persistent epithelial defects (PED), corneal stroma scarring, or swelling [1]. We report a case of an oncologic patient who developed NK after completing radiation therapy for treating a pontocerebellar angle and trigeminal ganglion metastases. Our purpose is to describe an unusual case of trigeminal neurotrophic cornea ulcer (TNCU) after a damage to the third nerve and its ganglion.

## Case presentation

**Figure 1:**
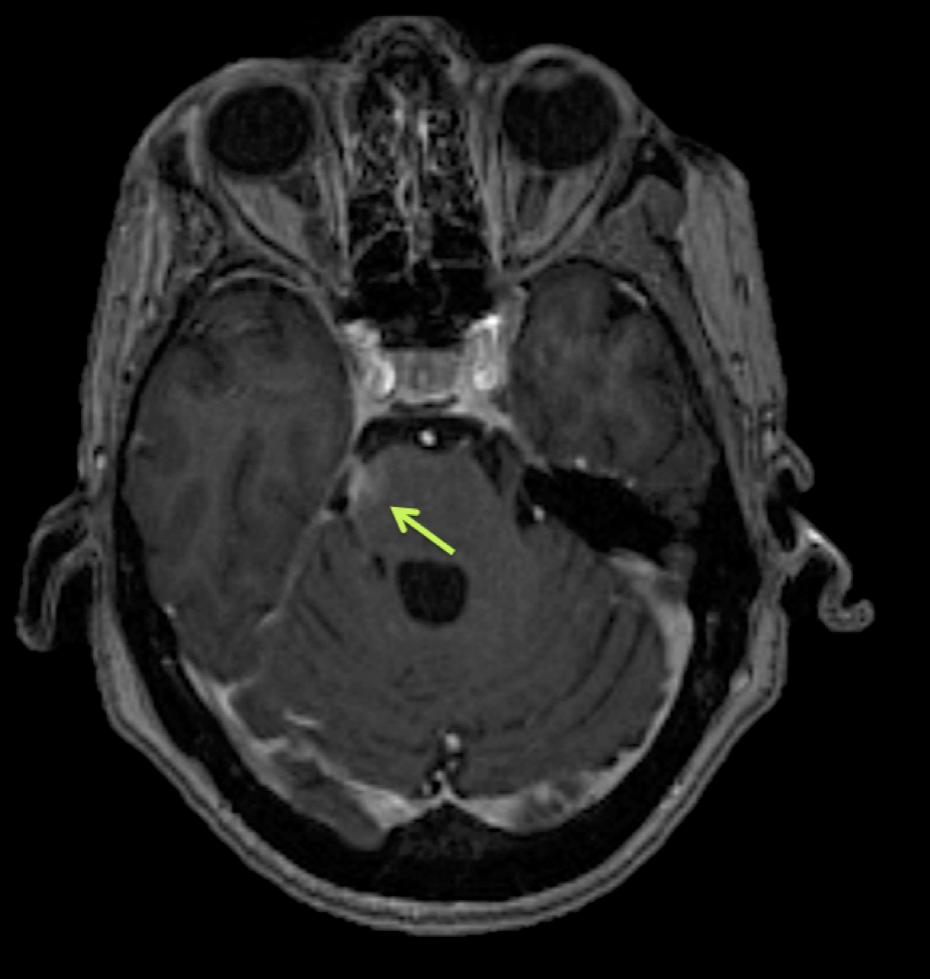
Brain MRI Post-Gadolinium axial T1-weighted image showing a right paramedian pontine area of contrast enhancement, at the level of the emergence of the fifth cranial nerve (green arrow).

A 63-year-old Caucasian female was referred to the cornea service of our clinic complaining about blurry vision for about one month. Topical broad-spectrum antibiotic drops were previously prescribed elsewhere for the cornea ulcer she had in her right eye, without improvement. Our first visual examination revealed a loss of superficial corneal stroma of 5.5 mm diameter, and staining with fluorescein indicated a 9 mm diameter corneal disepithelization. We evaluated corneal sensitivity by using a corneal Cochet-Bonnet contact aesthesiometer and it showed a decreased result. Moreover, the corneal reflex was absent and lacrimation was decreased in her right eye. The left eye was within normal limits, with no previous history of eye diseases. Her medical record showed arterial hypertension, anxiety, and breast cancer complicated by brain metastases. Cancer-related left brachial plexopathy, and progressive spastic paraparesis due to spinal cord compression occurred secondary to metastatic brain infiltration (Figure 1). In July 2009, she underwent a right simple mastectomy with a right axillary lymph node dissection (ALND). Pathology had confirmed an invasive ductal carcinoma, moderately differentiated, 2.4 cm, estrogen/progesterone receptor negative, HER2 positive +3, with negative surgical margins. Three out of the eight right axillary lymph nodes dissected, including the sentinel lymph node, were positive.

**Figure 2:**
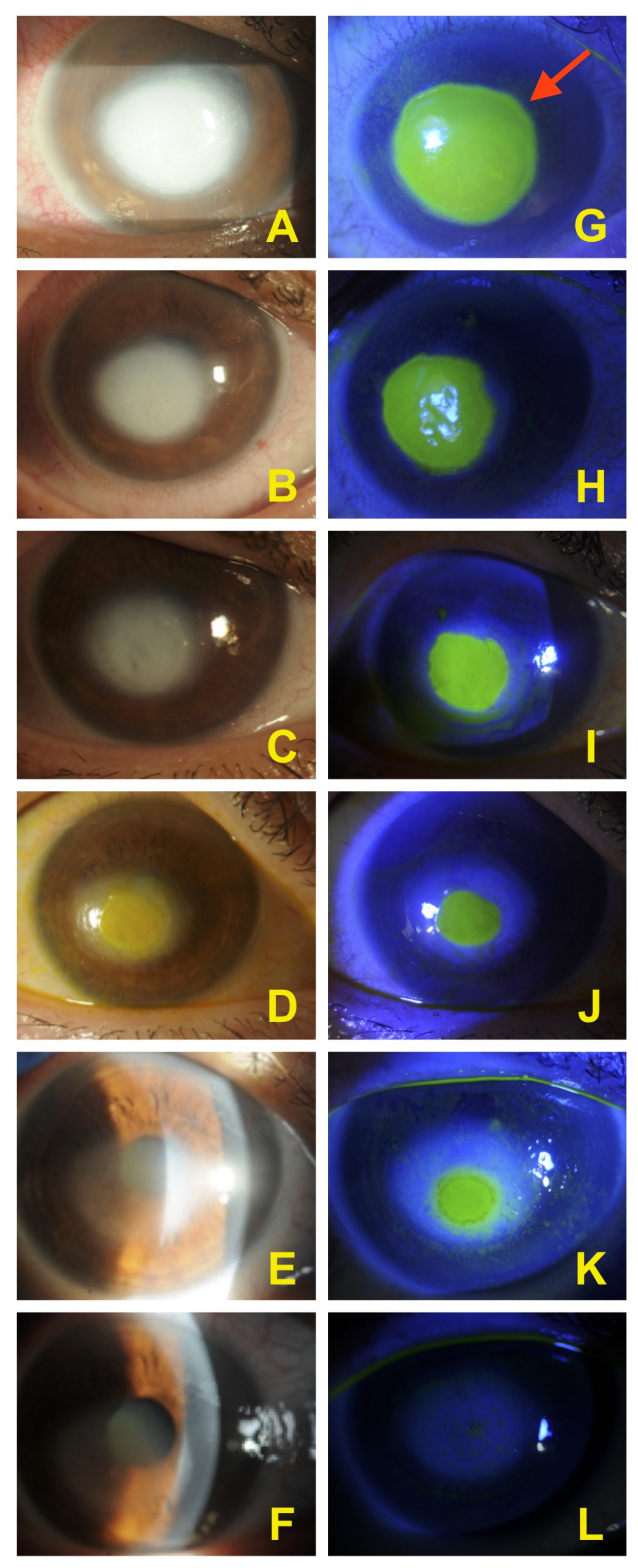
Corneal healing with autologous serum Corneal improvement from day one to day 15 with autologous serum tears with reduction of the stromal opacity (pictures A to F). Fluorescein stains corneal stroma but not intact corneal epithelium, thus demarcating the area of the epithelial loss (red arrow). Pictures G to K show progress in re-epithelialization. Complete re-epithelization is showed by picture L.

The standard of care for a patient with these tumor features is surgery plus adjuvant chemotherapy and immunotherapy [3]. Thus, in agreement with the National Comprehensive Cancer Network (NCCN) practice guidelines, combined treatment with trastuzumab and tamoxifen was started. After seven years of a disease-free interval, in March 2016, pontocerebellar angle and trigeminal ganglion metastases occurred. Thus, the patient was assigned to go through a local cytoreductive radiotherapy scheduled as 20 Gy in 5 fx delivered over one week. One month later, the patient developed a corneal ulcer. At that time, neither diabetes nor any other co-morbidities were present, and the patient was not taking any other medications. The treatment was started with eye patching and application of fluoroquinolone q2h and vitamin ointments with frequent monitoring on an outpatient basis every, one, two days pending improvement. The corneal ulcer improved after ten days, but it did not heal. A therapeutic contact lens was then applied but it was not sufficient, with no significant ocular improvement reported after seven days. Forty percent autologous serum tears (AST) every two hours was proposed. After obtaining a written informed consent, the patient was tested for blood-borne infections, including hepatitis B and C, syphilis, and HIV serology. The venipuncture was performed at the antecubital fossa under aseptic conditions to collect 100 ml of whole blood into sterile containers. This was left standing for two hours to ensure complete clotting. The blood was then centrifuged for 15 minutes at 3000 RPM. The serum was then separated in a sterile manner and diluted to 40% using saline solution. In approximately 15 days, the cornea markedly ameliorated with combined improved in visual acuity. Visual acuity improved from hand motion to 20/40 (Figure 2). After the treatment, residual corneal scarring remained in her right eye.

## Discussion

In our case, the underlying cause of the neurotrophic keratopathy seems to be the cornea denervation due to the previous trigeminal injury. Sensory nerves could affect the corneal epithelialization by releasing neuro-mediators, such as acetylcholine, substance P, and calcitonin gene-related peptide. Their lack, resulting from a denervation, could determine an epithelial defect even in the absence of direct injury [1, 4].This can be triggered by a myriad of conditions [5]; in our case damage to the third nerve caused by metastases were probably the underlying cause of the disease. Radiotherapy delivered directly to the pontocerebellar angle might also have played a role, even if the occurrence of such radiation-induced damage is rare; the pathophysiological mechanisms are not yet fully understood, and usually, clinical signs appear several years afterward [6]. Recently, the use of AST has gained wide acceptance for the treatment of ocular surface disorders unresponsive to conventional medical therapy [7]. Such conditions include persistent epithelial defects or severe dry eyes [8]. The serum has biomechanical and biochemical properties like natural tears [9]. As described in our case and accordance with the current literature, treatment with AST is safe and no substantial side effects have been reported [5]. Its neurotrophic support can be explained by its content: epithelial and neurotrophic growth factors as vitamin A, fibronectin, epidermal growth factor, transforming growth factor b (TGFb), substance P, and insulin-like growth factor-1 [10]. These essential components can contribute to healing in cases of PED usually associated with an already compromised ocular surface. We use a centrifugation rate of 3000 RPM for 15 minutes, and it results in good separation of serum and blood clot without inducing hemolysis. This g-force can help to yield a larger volume of serum from a full blood sample, and it can also result in a lower concentration of TGFb. The latter, if exceeded, can slow down the re-epithelialization [9]. The efficacy of AST seems to be both dose and concentration-dependent [9]. In our experience, 40% concentration has proved to be effective.

## Conclusions

Our report shows that brain metastasis should always be suspected if TNCU occurs in an oncology patient. Deeper neurotrophic ulcers can cause serious scarring and cornea perforation, leading to severe visual impairment; a rapid diagnosis and prompt treatment are needed to prevent such complications. Topical medication with AST may be a useful therapeutic option for TNCU after a damage of the Gasser's ganglion.
